# 

**Published:** 1880-03

**Authors:** 


### HYMENEAL.

"Domestic happiness, thou only bliss
Of paradise that has survived the fall."

Judkins—Anderson.—In Cincinnati, O., at the residence of Mrs. M. W. Latham, No. 107 Broadway, February 10, 1880, by the Rev. G. H. Kinsolving, Dr. William Judkins and Miss Nellie Anderson.

---

### NATALITIAL.

"Of all the joys that brighten suffering earth,
What joy is welcomed like a new-born child?"

Born, at Arlington, Maryland, on the 7th of February, 1880, a son, to Dr. and Mrs. Charles G. Hill. We extend our congratulations to our friend and whilom pupil, on the birth of his second son; and the wish that he may live to be as good and true a man as his intelligent and generous father.

---

### NECROLOGICAL.

"Death is the crown of life:
Were death denied, poor man would live in vain."

Dr. H. H. Toland, a prominent physician of San Francisco, died on Friday, February 27, of apoplexy. He was a native of South Carolina.

Dr. Mordecai Lawrence, a well-known physician, died on Saturday, February 21, at Ardmore, Montgomery Co., Pa., aged seventy-seven years.

Dr. Henry M. Bullitt died at his residence, in Louisville, February 4. He was largely instrumental in founding the Kentucky School of Medicine in 1850, and the Louisville Medical College in 1870.

Dr. Charles H. Weikel, of Philadelphia, died in Florida on Monday, February 16.

